# Development of Compact High-Voltage Power Supply for Stimulation to Promote Fruiting Body Formation in Mushroom Cultivation

**DOI:** 10.3390/ma11122471

**Published:** 2018-12-05

**Authors:** Katsuyuki Takahashi, Kai Miyamoto, Koichi Takaki, Kyusuke Takahashi

**Affiliations:** 1 Faculty of Science and Engineering, Iwate University, Morioka, Iwate 020-8551, Japan; g0317144@iwate-u.ac.jp (K.M.); takaki@iwate-u.ac.jp (K.T.); 2 Agri-Innovation Center, Iwate University, Morioka, Iwate 020-8550, Japan; 3 Morioka Forest Association, Morioka, Iwate 028-4132 Japan; sotoyama@smile.ocn.ne.jp

**Keywords:** pulse power, electrical stimulation, electric field, mushroom, *L. edodes*, *Lyophyllum deeastesSing*

## Abstract

The compact high-voltage power supply is developed for stimulation to promote fruiting body formation in cultivating *L. edodes* and *Lyophyllum deeastes* *Sing.* mushrooms. A Cockcroft-Walton (C-W) circuit is employed to generate DC high-voltage from AC 100 V plug power for the compact, easy handling and high safety use in the hilly and mountainous area. The C-W circuit is connected to high-voltage coaxial cable which works for high-voltage transmission and for charging up as energy storage capacitor. The output voltage is around 50 kV with several microseconds pulse width. The dimension and weight of the developed power supply are 0.4 × 0.47 × 1 m^3^ and 8.1 kg, respectively. The effect of the high-voltage stimulation on enhancement of fruiting body formation is evaluated in cultivating *L. edodes* and *Lyophyllum deeastes* *Sing.* mushrooms using the developed compact high-voltage power supply. The conventional Marx generator is also used for comparison in effect of high-voltage stimulation for fruiting body formation. *L. edodes* is cultivated with hosting to natural logs and the pulsed high voltage is applied to the cultivated natural logs. The substrate for *Lyophyllum deeastes Sing.* cultivation consists of sawdust. The results show that the fruiting body formation of mushrooms of *L. edodes* for four cultivation seasons and that of *Lyophyllum deeastes Sing.* for two seasons both increase approximately 1.3 times higher than control group in terms of the total weight. Although the input energy per a pulse is difference with the generators, the improvement of the fruit body yield mainly depends on the total input energy into the log. The effect for promotion on fruiting body formation by the developed compact high-voltage power supply is almost same that by the conventional Marx generator.

## 1. Introduction

The application of a pulsed high voltage to improve the yield in edible mushroom cultivation has also been attempted by some research groups. The fruiting capacity of shiitake mushroom (*L. edodes; L. edodes*) was remarkably promoted by applying a pulsed high voltage to log wood [1,2,3]. This effect was also recognized in *L. edodes* fruiting on a mature sawdust-based substrate [4,5]. The fruit body (sporocarp) yield in the electrically stimulated substrate was observed to be 1.7 times more than that in the spontaneous fruiting substrate control [6]. This effect was also recognized in the sporocarp formation of edible mushrooms: *Grifola frondosa*, *Pholiota nameko*, *Flammulina velutipes*, *Hypsizygus marmoreus*, *Pleurotus ostreatus*, *Pleurotus. eryngii* and *Agrocybe cylindraceas* [7,8,9]. Sporocarp yield, that is, fruit body formation in the electrically stimulated substrate, was observed to be 130–180% greater than that in the spontaneous fruiting substrate control [6]. The pulsed high-voltage stimulation technique was also applied to ectomycorrhizal fungi, which form associations with some types of wood, such as *Laccaria laccata* and *Tricholoma matsutake* [9,10].

Many types of electrical power supplies have been employed to provide electrical stimulation. A large-scale 1 MV high-voltage impulse generator was used to stimulate *L. edodes* log wood [1]. High-voltage AC was used to stimulate an *L. edodes* sawdust substrate [4]. Inductive energy storage (IES) pulsed power generators have favorable features for mushroom-cultivating applications, for example, they are compact, cost effective, light and have high voltage amplification compared with capacitive energy storage generators such as the impulse generator [9,10]. The yield of *L. edodes* fruiting bodies was improved with high-voltage stimulation generated by the IES pulsed power generators [2,3]. The effect of the pulsed voltage stimulation on some other types of mushroom such as *P. nameko* and *Lyophyllum decastes* (*L. decastes*) was also confirmed using an IES generator developed for the improvement of mushroom yield [6,7]. As a result of these studies, the total harvested weight from log wood and/or sawdust substrates for mushroom cultivation increased by applying a pulsed voltage as an electrical stimulation.

The hilly and mountainous area is suitable for the farmland of mushroom production because of its abundant forest resources and the significant overnight temperature changes. The method of pulsed voltage stimulation has been attracting attention as a promising technology that replaces the conventional stimulation methods such as the immersing water and the beating mushroom logs and improve the working efficiency in the hill and mountains. On the other hands, the electrical power supplies for pulsed voltage stimulation, such as Marx and IES pulsed power generators, has a heavy weight, a large size and a low safety because of its high power, large charging energy and high voltage, which is the major obstacle in a practical use. In this study, a Cockcroft-Walton (C-W) circuit is developed and employed to generate DC high-voltage from AC 100 V plug power as a compact and easy-handling high-voltage power supply for pulsed voltage stimulation. The promotion of mushroom production is affected by electric parameters such as applied voltage, pulse width and input energy. In the present experiment, the influence of the electric parameters on the mushroom production is evaluated using two types of power supply, C-W circuit and a conventional Marx generator [11]. The experiments are conducted on the mushroom production using two different fruiting types, Shiitake (*L. edodes*) mushroom and Hatakeshimeji (*Lyophyllum deeastes Sing.*) mushroom. The mushrooms are cultivated at a farmland in the hilly and mountainous area.

## 2. Experimental Setup

### 2.1. Pulsed Power Generators

**Figure 1:**
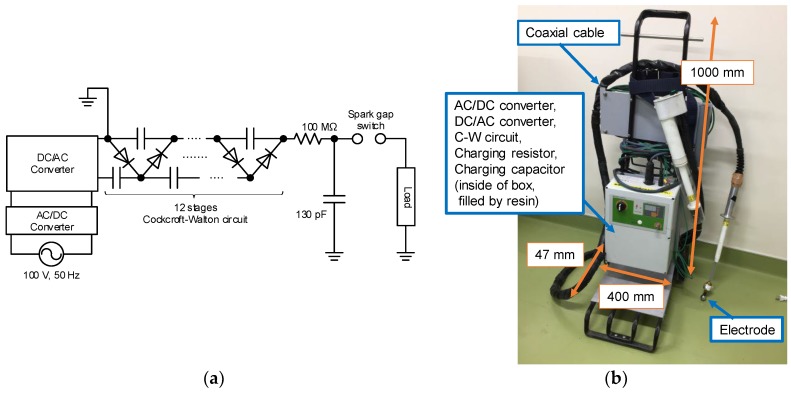
Circuit diagram (**a**) and photograph (**b**) of C-W circuit.

**Figure 2:**
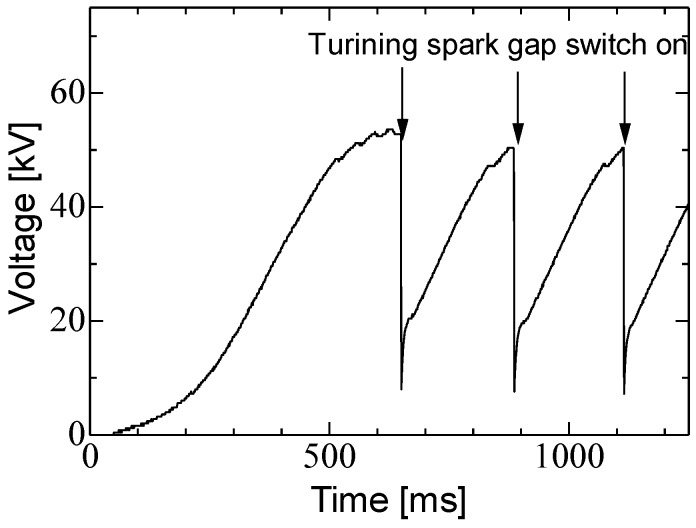
Waveforms of output voltage of C-W circuit during charging without load.

Figure 1 shows circuit diagram and photograph of high voltage pulsed power supply based on Cockcroft-Walton circuit (Green techno, Kanagawa, Japan; GM100) [12,13]. The circuit is consisted of an AC/DC converter, a DC/AC converter, 12 stages of ceramic capacitors and diodes, a charging capacitor, a 100 MΩ charging resistor and a spark gap switch. The ceramic capacitors have a capacity of several hundred pF. The DC/AC converter consists of a high voltage transformer driven by a resonance circuit and its output voltage of DC/AC converter is 6.2 kV with frequency of 25 kHz. The charging capacitor consists of a 2.6 m coaxial cable with the capacitance of 130 pF (50 pF/m). The AC/DC and DC/AC converters, C-W circuit, the charging capacitor and the charging resistor are inside of the box as shown in Figure 1b, which is filled by a resin for insulation. Figure 2 shows the charging voltage to the charging capacitor. Although the charging time depends on number of the stages and the frequency, the capacitor is charged during approximately 230 ms after turning the spark gap switch on because the output current of the DC/AC converter is limited.

**Figure 3:**
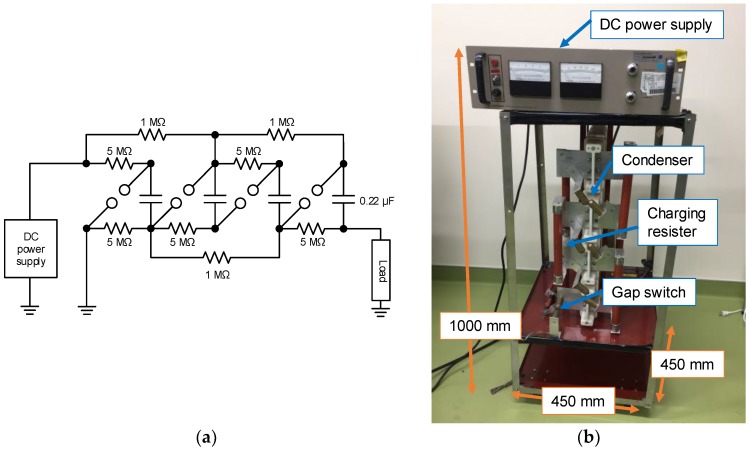
Circuit diagram (**a**) and photograph (**b**) of Marx generator.

Figure 3 shows the circuit diagram and photograph of pulsed power generator based on Marx generator [8,11]. The Marx generator consists of 4 energy storage 0.22 μF capacitors (Maxwell, 31160), charging resistors (1 and 5 MΩ) connected to the capacitors and the spark gap switches. The capacitors are charged up using a high voltage DC power supply (Gamma high voltage research, RR3-5R/100) up to 12.5 kV. The charging time is required for approximately 10 s because of the output current limit. After charging up the capacitors, a spark gap switch is manually closed. When a spark gap switch is closed, the other switches are sequentially closed automatically and the connection of capacitors is changed from parallel to series. The voltage is stepped up and is applied to the load.

Although the sizes of the Marx generator (1.0 m × 0.45 m × 0.45 m) and C-W circuit (0.4 m × 0.47 m × 1.0 m) are almost same; however, the weights of them are 39.4 kg and 8.1 kg, respectively. Therefore, the handling of C-W circuit in the farmland in hilly and mountainous areas is much easier than that of Marx generator.

### 2.2. Electrical Stimulation to L. edodes

**Figure 4:**
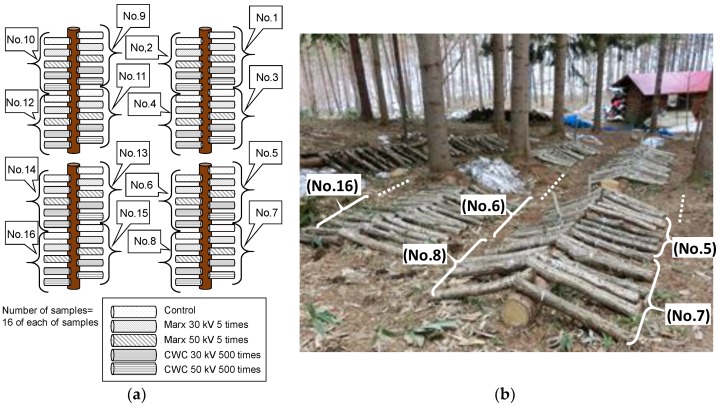
Arrangement (**a**) and photograph (**b**) of *L. edodes* logs for cultivation.

The cultivating mushroom, *L. edodes*, is inoculated on natural logs of *Quercus crispula Blume* two years before the experiment. The strain of the fruiting type is Mori#290 (Mori. Co. Ltd., Gunma, Japan). The dimensions of shiitake mushroom logs with a length of 0.9 m and a diameter of about 0.1 m. The logs are covered with a blackout curtain to maintain the moisture content in the logs hosting the mushroom hyphae. After two years incubation, the blackout curtain is unveiled and the logs are placed side by side under environment as shown in Figure 4. 

Mushroom fruits body production varies among logs, which makes the evaluation difficult. Therefore, it is needed to reduce the influence of variation on the evaluation. In the experiments, the total 80 logs are divided into 4 pulsed voltage stimulated groups and a control group without pulsed voltage to make the average amount of mushroom production of each group almost same after 1st flash. The number of logs for each stimulated group and a control group is 16 logs and numbered from 1 to 16. After the 1st flash, the logs are alternately rearranged as shown in Figure 4a to reduce the influence of arrangement positions.

**Figure 5:**
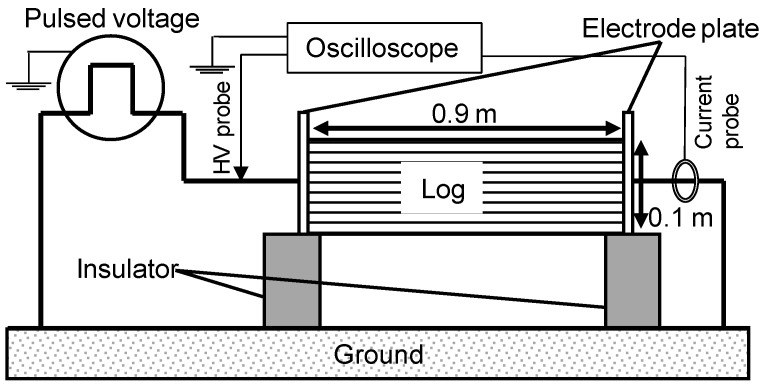
Experimental setup for pulsed voltage stimulation to the *L. edodes* logs.

The pulsed voltage is applied to the logs 1 month before the date that mushroom fruit body is usually expressed. Since the impedance of the logs is affected by the moisture content of wood, the pulsed voltage is applied when that day and its previous day are not rained. The fruit body of mushrooms can be cropped from the logs in every two seasons, spring and autumn, over two years. Therefore, the experiments are conducted for 4 seasons, from 15 May to 20 June in 2017 (1st flush), from 22 September to 22 November in 2017 (2nd flush), from 4 April to 11 June in 2018 (3rd flush) and from 17 September to 9 November in 2018 (4th flush). Figure 5 shows the experimental setup for pulse application to the logs. To apply the pulse voltages to logs, the electrode plate was installed at both ends of logs placed on an insulator of acrylic. The pulsed voltages are applied to the logs at the first day of 2nd and 4th flush seasons using the C-W circuit and the Marx generator. The total input energy into the logs is controlled by the amplitude and the number of the applying. Four groups are stimulated by the pulsed voltages with the different amplitudes, 30 kV and 50 kV, for each generator. The number of pulses is fixed at 500 times in the case of C-W circuit and 5 times in the case of Marx generator. Because the mechanical stress to the mushroom hypha could be affected to the mushroom production, the logs in the control group are set the experimental setup without the applying voltage. The fruit bodies of mushroom are cropped when their pileus is 80% opened, which is suitable to be in the market.

### 2.3. Electrical Stimulation to Lyophyllum deeastes Sing

**Figure 6:**
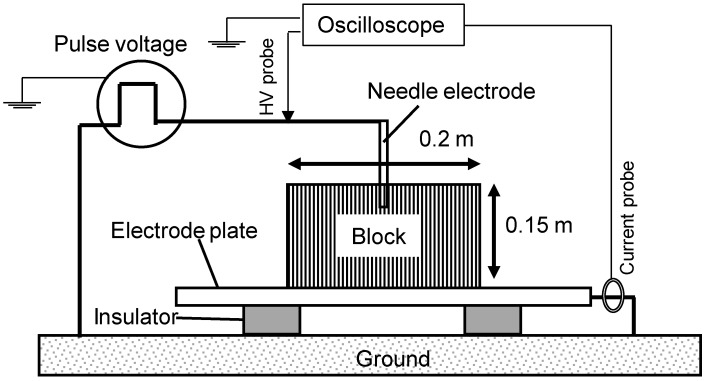
Experimental setup for pulsed voltage stimulation to the *Lyophyllum deeastes Sing.* sawdust block.

The substrate for *Lyophyllum deeastes Sing.* cultivation consists of sawdust from *Cryptomeria japonica* produced by Kamiyotsuba agricultural cooperative (Kami, Miyagi, Japan). The strain of the fruiting type is Miyagi LD-2 (Tsukidate bio service. Co. Ltd., Miyagi, Japan). The dimensions of the sawdust substrate are 0.12 m × 0.2 m × 0.1 m and it has a cuboid-block shape. The weight of the substrate was 2.5 kg ± 200 g. *Lyophyllum deeastes Sing.* fungus are inoculated on the block and the incubated for 50–60 days under the temperature of 22–23 deg-C with a relative humidity of 65–70%. The blocks are stimulated by the pulsed voltage after the incubation. The pulsed voltage was applied to a needle electrode with a 4 mm diameter driven into the block to a depth of 50 mm, as shown in Figure 6, using C-W circuit. The total input energy into the blocks are controlled by the amplitude and number of the applying voltage. Four groups are stimulated by the pulsed voltages with the different amplitudes, 30 kV and 50 kV and the different numbers of pulses, 100 times and 500 times. The number of blocks for each group is 16 and numbered from 1 to 16.

**Figure 7:**
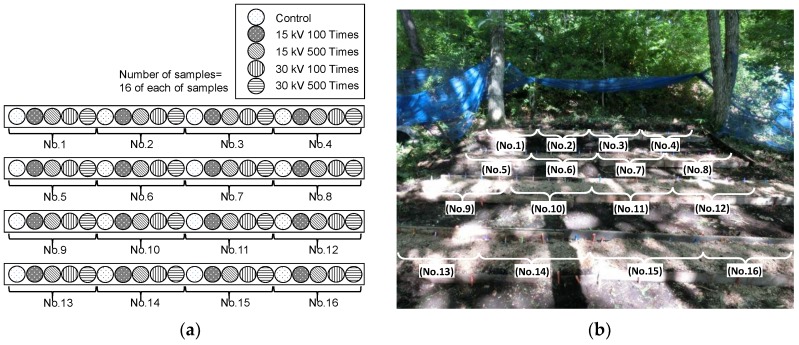
Arrangement (**a**) and photograph (**b**) of the *Lyophyllum deeastes Sing.* sawdust block for cultivation.

After the stimulation, the blocks are buried under the soil with the unburied upper surface as shown in Figure 7. The blocks are alternately arranged as shown in Figure 7a to reduce the influence of the arrangement positions. The fruit bodies of mushroom are cropped when their pileus is 80% opened, which is suitable to be in the market.

## 3. Results

### 3.1. Electrical Stimulation to Logs and Cropping Fruits Body of L. edodes

**Figure 8:**
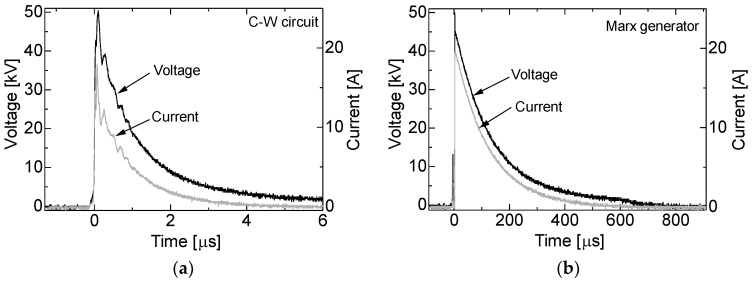
Typical waveforms of applied voltage and current to the *L. edodes* logs using (**a**) C-W circuit and (**b**) Marx generator.

Figure 8a,b shows the typical waveforms of the applied voltage and output current to the shiitake mushroom logs using the C-W circuit and the Marx generator in 4th flush season. When the gap switch of the circuits is shortened, the voltage charged at the capacitors is applied to the log and then the voltage exponentially decays. The impedance of the log is calculated from the waveforms and is 2.67 kΩ with a standard deviation of 0.64 kΩ in 2nd flush season and 5.29 kΩ with a standard deviation of 1.97 kΩ in 4th flush season. The differences of the impedance could be caused by the moisture contents of the logs and a decay with a hypha filled in the log. The time constant in the cases of C-W circuit and Marx generator in the case of 2nd flush season are approximately 1.2 μs with a standard deviation of 0.28 μs and 190 μs with a standard deviation of 51 μs, respectively and those in the case of 4th flush season are approximately 150 μs with a standard deviation of 0.61 μs and 410 μs with a standard deviation of 0.41 ms, respectively. Although the impedances and the time constants are difference, the total input energy into the logs are almost same in the two seasons. In the case of C-W circuit, high voltage pulses with maximum voltage of 30 kV and 50 kV are applied for 500 times and the total input energy are 60 J and 148 J, respectively. In the case of Marx generator, the high voltage pulses with maximum voltage of 30 kV and 50 kV are applied to the cultivation log for 5 times and the total input energy are 127 J and 345 J, respectively. Assuming that the electric field in the log is uniform, the electric field inside log in the case of 30 kV and 50 kV is 34 and 56 kV/m, respectively. 

**Figure 9:**
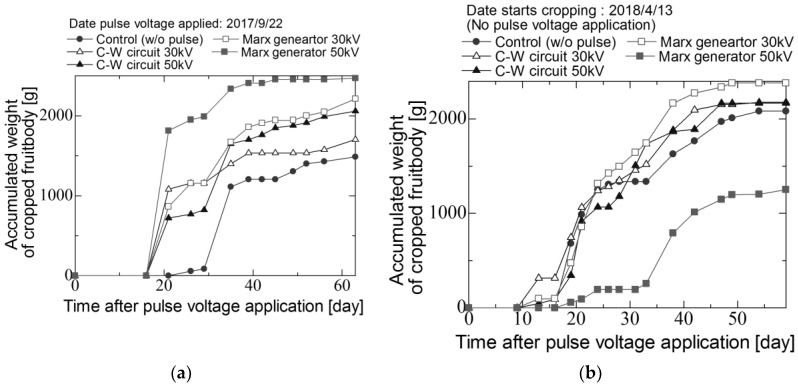

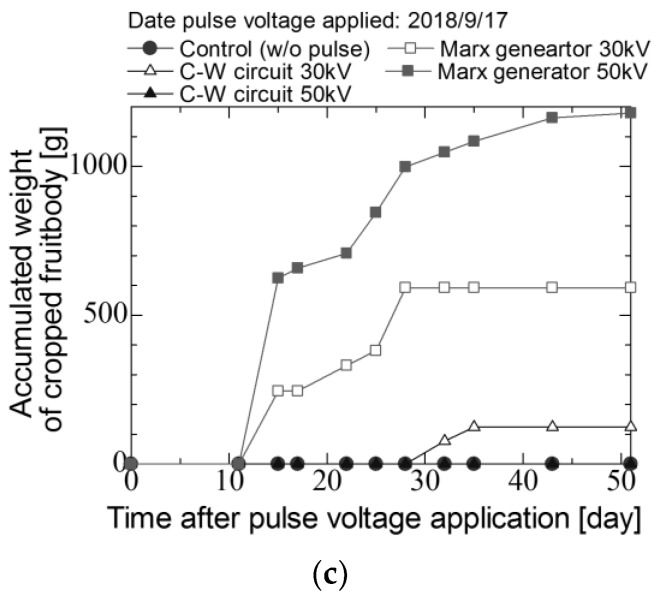
Diurnal change of the accumulated weight of fruitbody of *L. edodes* in (**a**) 2nd flush, (**b**) 3rd flush and (**c**) 4th flush.

Figure 9a–c shows the diurnal change of the accumulated weight of fruitbody of shiitake mushroom in three seasons, 2nd flush, 3rd flush and 4th flush. In the 2nd flush (Figure 9a), the accumulated of fruit body in the case of applying voltage is higher than that in the control group with the harvest duration. The yield of fruit body in the cases of the stimulate groups of 30 kV and 50 kV using the C-W circuit and 30 kV and 50 kV using the Marx generator are 1.15, 1.38, 1.49 and 1.66 times higher than the control group. In the 3rd flush, the pulsed voltages are not applied to logs (Figure 9b), the yield of fruit body does not increase in the cases of stimulated groups in comparison with 2nd flush. The yield of fruit body in the cases of the stimulated groups of 30 kV and 50 kV using the C-W circuit and 30 kV using the Marx generator are 1.04, 1.14 and 1.04 times higher than the control group in the 3rd flush. The yield in the case of the stimulated group of 50 kV using the Marx generator is much lower than other groups. Generally, the yield depends on the yield in previous flush, which could cause the decrease of the yield. In the 4th flush, the fruit bodies are cropped from only the stimulated groups of 30 kV using C-W circuit and 30 and 50 kV using the Marx generator.

**Figure 10:**
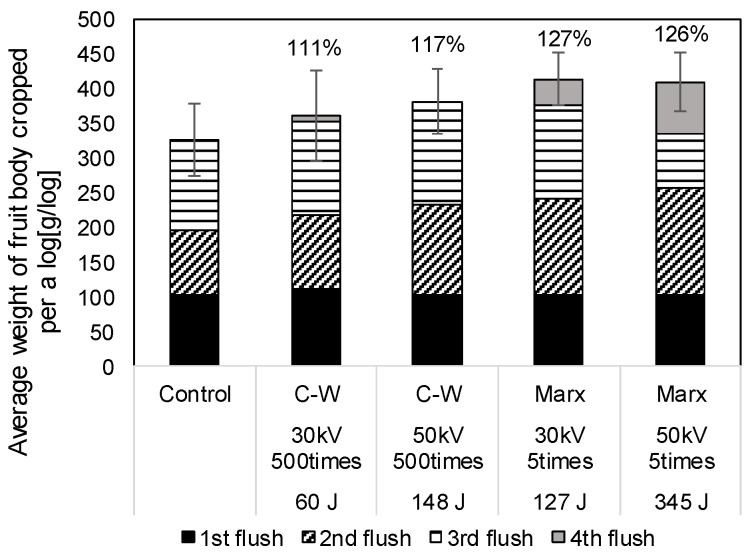
Average yield of fruit body fruitbody of *L. edodes* per a log for 4 flushes.

Figure 10 shows the average weight of fruit body cropped per a log, cropped for 4 seasons. The error bars represent the standard error. Since the logs are divided into 5 groups stimulated groups after 1st flash, the amount of the weight of fruit body is almost same. The average weight of fruit body is improved by applying pulse voltages and increased with increasing total input energy into the log. The average weight of fruit body in the case of the Marx generator is approximately 1.3 times higher than that in the control group. The results show that the improvement of the fruit body yield mainly depends on the total input energy into the log.

### 3.2. Electrical Stimulation to Blocks and Cropping Fruits Body of Lyophyllum deeastes

**Figure 11:**
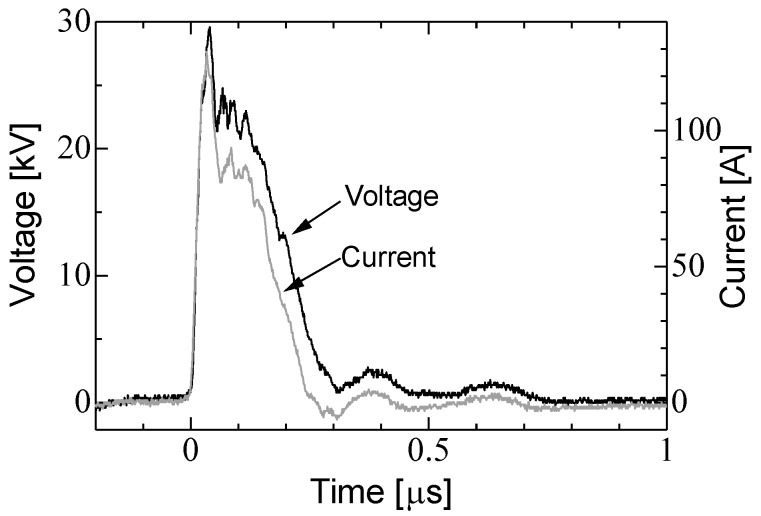
Waveforms of applied voltage and current to the *Lyophyllum deeastes Sing.* Sawdust block using. C-W circuit.

**Figure 12:**
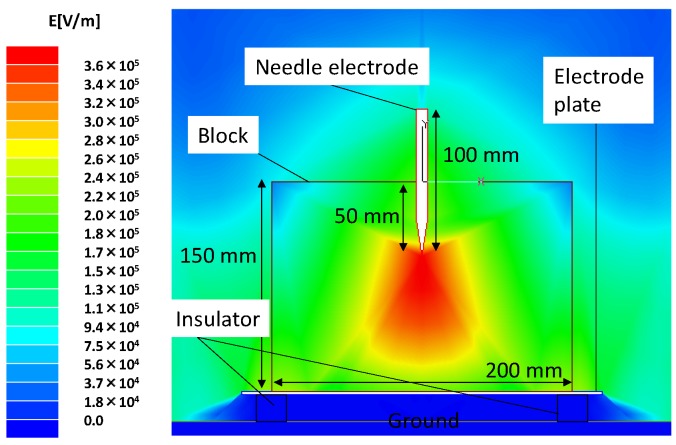
Electric field distribution inside of *Lyophyllum deeastes Sing.* Sawdust block for applied voltage of 30 kV.

Figure 11 shows waveforms of applied voltage and output current to the *Lyophyllum deeastes* mushroom block. The resistivity of the bed is 45 Ωm and the impedance of the block is calculated from the waveforms and is 0.35 kΩ with a standard deviation 0.12 kΩ. Because the coaxial cable in the C-W circuit acts as a transmission line, the waveforms of applied voltage and output current are distorted and do not have an exponential shape by the forward and backward transmitted waves [14]. Figure 12 shows the electric field distribution analyzed by the finite element method (Ansoft Maxwell 2D). The analysis results show that the electric field inside of the block is concentrated at the tip of the needle and is ranged from 18 to 360 kV/m with the applied voltage of 30 kV. The input energy per a pulse in the cases of 15 and 30 kV applied voltages are 54 mJ and 27 mJ, respectively. The total input energy in the case of 15 kV is 5.4 J for 100 times pulses and 27 J for 500 times pulses and that of 30 kV is 27 J for 100 times pulses and 160 J for 500 times pulses.

**Figure 13:**
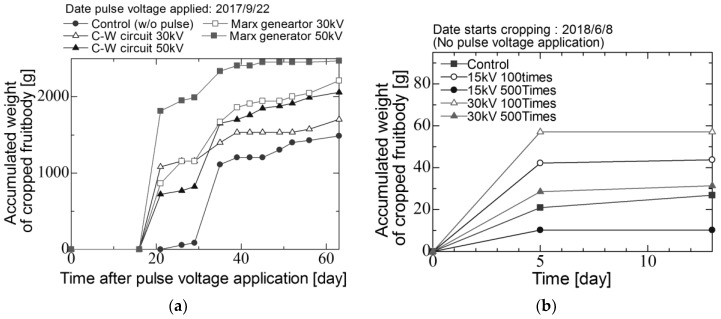
Diurnal change of the accumulated weight of fruitbody of *Lyophyllum deeastes Sing* in (**a**) 1st flush and (**b**) 2nd flush.

**Figure 14:**
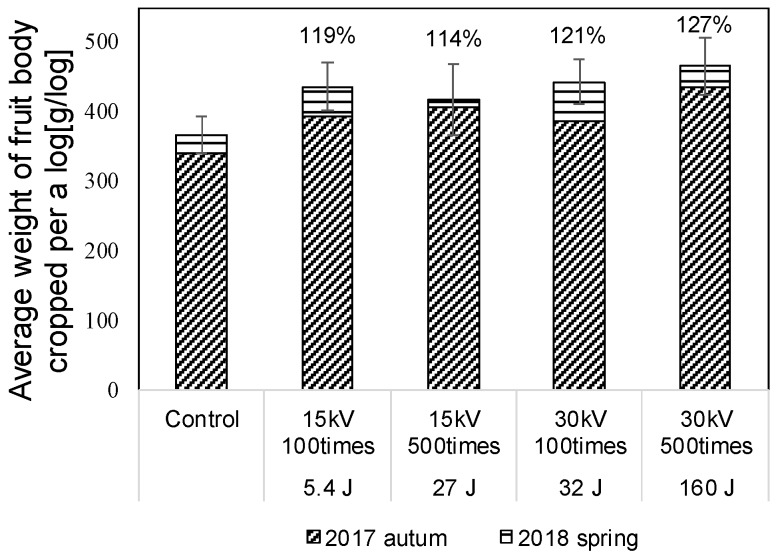
Average yield of fruit body fruitbody of *Lyophyllum deeastes Sing* per a log for 2 flush seasons.

Figure 13a–c shows diurnal change of accumulated weight of fruitbody of *Lyophyllum deeastes* mushroom from 27 August to 25 October in 2017 and from 13–21 June in 2018. The pulsed voltage is applied to the logs at the first day using the C-W circuit. Figure 14 shows the average weight of fruit body cropped per a block for two flush seasons. The error bars represent the standard error. The average weight of fruit body was improved by applying pulse voltages and increased with increasing total input energy into the block. 

## 4. Discussion

When the pulsed voltages are applied to the logs, the mushroom hyphae are subjected to an electric field. When the frequency component of the applied pulse voltage is less than several MHz, the membrane of the cell, rather than the inside of the cell, is mainly subjected to the electric field [15]. The hyphae are accelerated and displaced according to the electric field by the electrostatic force such as a Coulomb force [16], which could induce a physical stress on the hyphae. It has been suggested that some genes encoding enzymes such as laccase and protease [17,18,19] could be upregulated by the physical stress [1,5] in the same manner as other physical stresses such as scrapping of surface hyphae, which induces fruit body formation [16,20]. Since the physical stress relates to the fruit body formation, the flush is accelerated and the amount of cropped fruitbody is increased in the seasons that the logs are stimulated by the voltage pulses as shown in Figure 10. 

The total *L. edodes* mushroom yield cropped from the logs is improved approximately 1.3 times from the control group by stimulating. The *Lyophyllum deeastes Sing*. mushroom cropped from the logs is also improved about 1.2 times. It has been reported that *Lyophyllum deeastes Sing*. yield is improved as same level using Marx-IES circuit. These results show the effect for promotion on fruiting body formation by the developed compact high-voltage power supply is almost same that by the conventional Marx generator.

In the economic aspect, the production improvement of 1.2 to 1.3 times using electric stimulation directly increases the farmer’s income. The electric power consumption of the high voltage pulsed power supply based on C-W circuit for the operation of the electrical stimulation is measured using an electric power monitor (SANWA SUPPLY, TAP-TST7) and is less than 40 W, which shows that the energy cost is low enough to be negligible. The time cost for the operation and the work load of the electrical stimulation could be much lower than the traditional stimulation methods such as a beating and a shaking. Furthermore, the acceleration of the flush as shown in Figure 9a and Figure 13a could reduce the total time cost for a cropping period, which could enhance the work efficiency. Therefore, the electrical stimulation has a high potential for the farmer’s management improvement.

## 5. Conclusions

The C-W circuit is developed and employed to generate DC high-voltage from AC 100 V plug power as a compact and easy-handling high-voltage power supply for pulsed voltage stimulation. The influence of the electric parameters on the mushroom production is evaluated using two types of power supply, C-W circuit and a conventional Marx generator. The weight of the C-W circuit is approximately 5 times lower than the Marx generator. The handling of C-W circuit in the farmland in hilly and mountainous areas is much easier than that of Marx generator. The experiments are conducted on the mushroom production using two different fruiting types, Shiitake (*L. edodes*) mushroom and Hatakeshimeji (*Lyophyllum deeastes Sing.*) mushroom. The fruiting body formation of mushrooms of *L. edodes* for four cultivation seasons and that of *Lyophyllum deeastes Sing.* for two seasons both increase approximately 1.3 times higher than control group in terms of the total weight. Although the input energy per a pulse is difference with the generators, the improvement of the fruit body yield mainly depends on the total input energy into the log. The effect for promotion on fruiting body formation by the developed compact high-voltage power supply is almost same that by the conventional Marx generator.
